# ZBTB24 (Zinc Finger and BTB Domain Containing 24) prevents recurrent spontaneous abortion by promoting trophoblast proliferation, differentiation and migration

**DOI:** 10.1080/21655979.2021.2019655

**Published:** 2022-01-17

**Authors:** Haibo Ruan, Zhenzhen Dai, Jinyu Yan, Xiaoxi Long, Yi Chen, Youlin Yang, Qian Yang, Jun Zhu, Meiyun Zheng, Xiahui Zhang

**Affiliations:** Department of Obstetrics and Gynecology, The First People’s Hospital of Wenling, Wenling, Zhejiang, China

**Keywords:** Recurrent spontaneous abortion (RSA), ZBTB24, trophoblast, epigenetic regulation

## Abstract

Recurrent spontaneous abortion (RSA) is a common complication during early gestation, which is associated with aberrant DNA methylation. Zinc Finger and BTB Domain Containing 24 (ZBTB24) plays a critical role in facilitating DNA methylation and cell proliferation. However, the regulatory role of ZBTB24 on trophoblast development in RSA remains unclear. In this study, ZBTB24 expression was compared between decidua tissues of RSA patients and induced abortion controls from a published dataset, which was further validated in placental villi tissues by RT-qPCR and Western blot. The roles of ZBTB24 in trophoblast proliferation, differentiation, and migration were investigated by functional assays after ZBTB24 knockdown or overexpression in HTR-8/SVneo cells. Our results showed that ZBTB24 expression was significantly decreased in RSA patients, and ZBTB24 expression level positively regulated cell viability, differentiation, and migration in HTR-8/SVneo cells. We further demonstrated that ZBTB24 modulated the expression of E-cadherin by altering the DNA methylation at the promoter region. Overall, the downregulation of ZBTB24 is implicated in RSA by inhibiting trophoblast proliferation, differentiation, and migration. Therefore, ZBTB24 may serve as a promising therapeutic target and diagnostic marker for RSA.

## Introduction

Recurrent spontaneous abortion (RSA) is a common clinical complication of early gestation with more than two consecutive spontaneous abortions before 20 weeks’ pregnancy [1–3]. It affects about 1% of fertile couples worldwide and increased incidence is observed in females approaching menopause [4]. Although parental abnormalities of chromosome, anatomic anomalies of uterus, autoimmune diseases, infections, and endocrine imbalance during gestation have been recognized as potential pathogenic factors for RSA, the etiology of nearly 50% RSA patients remains enigmatic [5,6].

Trophoblasts are crucial components in both placental and fetal development during early conception [7]. Impaired function of trophoblasts is associated with the abnormal uterine spiral artery rebuilding, which contributes to pre-eclampsia, intrauterine growth retardation and RSA [7,8]. The differentiation of placental trophoblasts from an anchorage-dependent epithelial phenotype into the mesenchymal-like invasive extravillous trophoblast (EVT) is crucial in the development of the maternal–fetal interface [9]. Understanding molecular mechanisms underlying this epithelial–mesenchymal transition (EMT) process could provide insights into the pathogenesis of RSA.

As a well-characterized epigenetic modification, maintaining parental DNA methylation and establishing *de novo* DNA methylation pattern is crucial for mammalian cell reprogramming and differentiation during pregnancy and fetal development [10–12]. Abnormal DNA methylation may yield karyotypically normal miscarriage, which is predominantly reported in women suffering from RSA [11,12]. ZBTB24 (zinc finger- and BTB domain-containing 24), a member of the Bric-à-brac/poxvirus and zinc finger (BTB/POZ) family, is a zinc finger transcription factor which coordinates with DNMT3B (DNA Methyltransferase 3 Beta) to control DNA methylation [13–15]. The downregulation of endogenous ZBTB24 significantly reduces B-cell proliferation [16]. However, its potential role in RSA has not been investigated.

In this study, through the bioinformatic analysis based on GSE113790 data set, we found that the expression level of ZBTB24 was significantly downregulated in tissues of RSA patients. Since the potential role of ZBTB24 in RSA has not been explored, we attempted to investigate whether ZBTB24 is implicated in regulation of EMT phenotype of trophoblast cells. Our results demonstrated that ZBTB24 expression level positively regulated cell viability, differentiation, and migration in HTR-8/SVneo cells. As an epigenetic regulatory factor, ZBTB24 modulated the expression of E-cadherin by altering the DNA methylation at the promoter region. Overall, our study suggests that the downregulation of ZBTB24 may contribute to the malfunction of trophoblasts in RSA by impairing the cell proliferation, differentiation, and migration.

## Materials and methods

### Public data set accession

The differently expressed genes of the decidua tissues between the samples with induced abortions (control group) and the patients with RSA (RSA) were obtained by analyzing the GEO dataset (GSE113790).

## Reagents

Trypsin-EDTA, pcDNA-3.1 plasmid, TRIzol reagent and Lipofectamine™ 3000 were purchased from Invitrogen (Rockville, USA). PrimeScript RT reagent kit and SYBR Premix Ex Taq II were purchased by TaKaRa (Dalian, China). Bicin choninic acid (BCA) Protein Assay Kit and β-hCG ELISA kit were supplied by Abcam (Cambridge, USA). Fetal bovine serum and RPMI medium were purchased from Gibco (Rockford, USA). RIPA Cell Lysis Buffer was obtained from Beyotime Biotechnology (Shanghai, China). SuperSignal Chemiluminescent HRP Substrate was purchased from Thermo Fisher scientific Inc (Rockford, USA). Antibodies against E-cadherin and GAPDH were from Cell Signaling Technology, Inc. (Danvers, USA). Transwell plate in the invasion assay was supplied by Corning Incorporated (New York, USA). Annexin V-FITC/PI apoptosis assay kit and protease inhibitor cocktail were from Beyotime (Nanjing, China). CCK-8 cell proliferation kit was purchased from Dojindo Corp (Kyushu, Japan).

## Placental villi tissue collection

The placental villi tissues were collected from RSA patients who had experienced more than two spontaneous abortions within 20 weeks of gestation (RSA group, n = 15), and non-RSA patients who underwent induced abortion (control group, n = 15). The placental villi tissues were snap-frozen in liquid nitrogen and stored in −80-degree deep freezer for subsequent analysis. The above procedures in this project have been approved by the Ethics Committee of the First People’s Hospital of Wenling.

## Cell culture

HTR-8/Svneo cells (human trophoblasts derived from 6 to 12 weeks gestation placenta) were provided by the Committee on Type Culture Collection of the Chinese Academy of Sciences (Shanghai, China) and cultured in RPMI 1640 medium with fetal bovine serum (FBS, 10%), penicillin (100 U/ml) and streptomycin (100 μg/ml), at 37°C in a humidified atmosphere with 5% CO_2_ and 95% air.

## Plasmids, siRNAs, and cell transfection

To overexpress ZBTB24, the full-length ZBTB24 cDNA was cloned into the pcDNA 3.1 plasmid (pcDNA3.1- ZBTB24), with empty pcDNA3.1 vector as the control. The siRNAs targeting ZBTB24 and control siRNA were synthesized by GenePharma (Shanghai, China) to knockdown ZBTB24. Cell transfection was performed using Lipofectamine® 3000 reagent (Thermo Fisher Scientific, L3000001). In 6-well plate, 60% confluent cells were transfected with 100 nM of siRNA or 6 ug of pcDNA3.1-ZBTB24 plasmid according to manufacturer’s instruction. Transfected cells were subjected to subsequent analysis 48 hours post-transfection.

## RNA purification and RT-qPCR

Trizol reagent was used to extract RNA from tissues (1 gram) and cells (1 million) according to the instructions. The extracted total RNA was dissolved in DEPC water and its concentration was measured with NanoDorp. 1 μg of total RNA was used for reverse-transcription into cDNA using PrimeScript RT kit following the guidance of the manufacturer. The resulted cDNA was diluted to 40 ng/μL, and the cDNA was used for qPCR assay with SYBR Premix ExTaq II kit on a Bio-Rad CFX 96 qPCR system (Richmond, CA, USA). The PCR cycling condition used: 95°C 2 mins, 40 cycles of 95°C 30 sec, 60°C 30 sec and 72°C 60 sec, with signal detection at the end of each cycle. Finally, the 2–∆∆Ct method was used to analyze the relative expression level and GAPDH was used as the internal reference gene. All primer sequences were synthesized and purchased from Shanghai Sangon Biotechnology Co., Ltd. (Shanghai, China): GAPDH: F_ ACAACTTTGGTATCGTGGAAGG; R_GCCATCACGCCACAGTTTC. ZBTB24: F_ ACTTGCTGCCAGTAGTGAATAC; R_ TGTGTAAGCCTTTACCAGGTCA. β-hCG: F_TGTTTAGTTTGATGGTATCGC; R_ ATACCCGAAACGATCCCC. E-cadherin: F_ CGAGAGCTACACGTTCACGG, R_ GGGTGTCGAGGGAAAAATAGG.

## Total protein separation and Western blotting assay

Total protein was extracted from cells (1 million) or 10 g of tissues using RIPA lysis buffer containing protease inhibitor cocktail (Thermo Fisher Scientific 78429, Waltham, MA, USA). Cells suspended in RIPA buffer were lysed on ice for 15 mins and lysed cells were centrifuged at 13,000 rpm for 10 mins. The supernatant containing total protein lysate was quantified by a BCA Protein assay kit (Beyotime P0009; Shanghai, China). 10 ug of total protein was used for SDS-PAGE electrophoresis on 8% gel. Separated protein in SDS-PAGE gel was transferred onto the PVDF membrane. After blocking with 5% skimmed milk for 1 hour, the membrane was then incubated with primary antibodies: ZBTB24 (1:1000, Themo Fisher Scientific # PA5-31622), E-cadherin (1:1000; Cell Signaling Technologies #3915), GAPDH (1:2000; Cell Signaling Technologies #2118) overnight at 4°C. The membrane was washed 3 times with TBST for 5 minutes each. After wash, the membrane was further incubated with HRP-linked secondary antibody (1:3000; Cell signaling #7074, MA, USA) at room temperature for 1 hour. Then the membrane was washed 4 times with TBST and the protein bands were visualized using an enhanced chemiluminescence kit (Santa Cruz, TX, USA, sc-2048) and photographed on the ChemiDoc MP imaging System (Bio-Rad, Hercules, CA, United States). The densitometry analysis was performed with Image J software (Bethesda, MD, USA).

## CCK-8 cell proliferation assay

Cells were seeded in to a 96-well plate at a density of 2000 cell/well and cultured in a humidified cell culture incubator for 0, 24, 48, and 72 hours, respectively. Subsequently, 10 μL CCK8 reaction solution was added to the cell culture at indicated time point and incubated for 1 hour in a humidified cell culture incubator. The light absorption value (OD value) in each condition was captured at 450 nm wavelength on a 680 Microplate Reader (Bio-Rad, Hercules, CA).

## Apoptosis analysis by flow cytometry

Cells with indicated treatments were trypsinized and washed twice with PBS, and resuspended in the staining solution. The detection of cell apoptosis was performed using the Annexin V-FITC/PI apoptosis assay kit (Beyotime, Nanjing, China) according to the manufacturer’s instructions. In brief, 5 μL Annexin V-FITC and 5 μL PI were added to the 1000 μL cell resuspension with 1 million cells and incubated for 20 mins in the dark. Stained cells were centrifuged and washed twice with PBS and resuspended in 400 μL PBS. The percentage of apoptotic cells was detected by a flow cytometry (Accuri C6, BD Biosciences, USA).

## Wound healing migration assay

Cells were seeded in 6-well plates for about 80% confluence. A scratch wound was created using a sterile 200 μL pipette tip in the central region of each well. The cells were incubated at 37°C for 24 h. Cell images were captured using an inverted light microscope (Leica AM6000 microscope). The migration distance was analyzed is using ImageJ software. The migration rate (closure rate) is calculated as ratio of would distance at 24 h/would distance at 0 h.

## Transwell migration assay

HTR-8/SVneo cells with different treatments were trypsinized and resuspended in serum-free medium. The transwell upper chamber (Corning, NY, USA, #3401) was used for migration assay, 5 × 10^5^ cells were inoculated into the transwell upper chamber in serum-free medium and 500 μL of 10% serum-containing medium was added to the lower chamber. After 24 hours, culture medium was discarded and the cells were fixed with 4% paraformaldehyde at room temperature for 10 mins and stained with 0.5% crystal violet (Sigma, Germany, #109218) for 20 mins. Cells were photographed under Leica AM6000 microscope (Leica, Wetzlar, Germany).and the number of invading cells was counted.

## ELISA

HTR-8/SVneo cells seeded in a 24-well plate (2 × 10^5^) were allowed to grow in 10% FBS containing media for 48 h. 100 μl of culture media was collected to determine the β-hCG (beta human chorionic gonadotropin) level. The β-hCG concentration was detected with a Human Chorionic Gonadotropin beta ELISA Kit (Abcam, ab178633) according to the manufacturer’s instructions.

## Chromatin immunoprecipitation (ChIP) assay

One million of cells were cross-linked by 1% formaldehyde, and the cross-linking was quenched by 0.125 M glycine. The nuclear chromatin was fragmented using a S220 Focused Ultrasonicator (Covaris, Woburn, MA, USA). Total fragmented chromatin (50 μg) was mixed with 5 μg anti-ZBTB24 (Thermo Fisher Scientific, # PA5-61860) or 10 μg anti-5-methylcytosine (5-mC) antibody (Abcam, ab21472) overnight at 4°C with rotation. The corresponding rabbit IgG was used as control. 10% of total fragmented chromatin was reserved as the input. The chromatin-antibody complex was then precipitated with Magna ChIP protein A + G magnetic beads (Millipore-Sigma). The beads were then washed sequentially with low-salt washing buffer (20 mM Tris, 2 mM EDTA, 50 mM NaCl, 0.01% SDS, and 1% SDS), high-salt washing buffer (same composition, 250 mM NaCl), and LiCl washing buffer (10 mM Tris, 1 mM EDTA, 250 mM LiCl, 1% NP40, and 1% sodium deoxycholate). The precipitated chromatin was eluted in 200 μl elution buffer (1% SDS, 100 mM NaHCO3 in dH_2_O). Cross-linking was reversed by adding 8 μl 5 M NaCl and incubating at 65°C overnight. RNase A (1 μl 10 mg/ml) was added to the tube for a 30 min incubation at 37°C, which was followed by the addition of 1 μl 20 mg/ml proteinase K for 2 h incubation at 42°C. Released DNA from both ChIP and input samples were purified with QIAquick PCR purification kit (Qiagen, Germantown, MD, USA). The DNA was quantified for the enrichment of ZBTB24 and 5-mC at E-cadherin promoter using the following primer (200 bp upstream of the start codon): P1: F_GTCAGTTCAGACTCCAGCCC, R_GAATGCGTCCCTCGCAAGTC.

## Statistical analysis

The measurement data are presented as mean ± standard deviation (SD). All the experiments were repeated three times. GraphPad Prism 8.0 (GraphPad, La Jolla, USA) was used for statistical analyses and data visualization. The statistical difference between two groups was compared using unpaired student’s t tests. Comparisons among multiple groups were analyzed using one-way analysis of variance (ANOVA) with Tukey’s post hoc test for pairwise comparison. Comparisons of data at multiple time points were examined using two-way ANOVA. Spearman correlation analysis was performed to determine the correlation between two genes. *p* < 0.05 indicates a statistical significance, * *p* < 0.05, **p < 0.01 and ***p < 0.001.

## Results

In this study, we found that ZBTB24 was significantly downregulated in tissues of RSA patients. We further attempted to investigate the implication of ZBTB24 in the regulation trophoblast phenotype. Our results demonstrated that ZBTB24 expression level positively regulated cell viability, differentiation, and migration in HTR-8/SVneo cells. As an epigenetic regulatory factor, ZBTB24 regulated the expression of E-cadherin by modulating the DNA methylation at the promoter region. Overall, our study suggests that the downregulation of ZBTB24 may contribute to the malfunction of trophoblasts in RSA by impairing the cell proliferation, differentiation and migration.

## ZBTB24 is downregulated in decidua and placental villi tissues of the RSA patients

**Figure 1.**
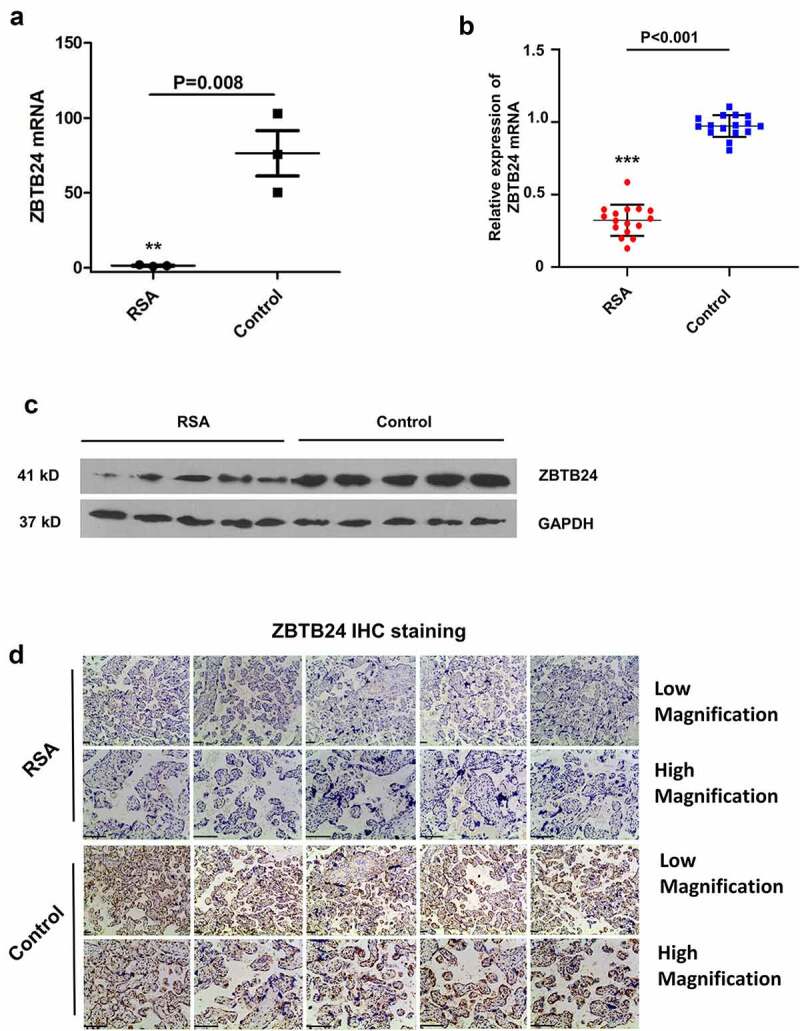
ZBTB24 expression level is significantly lower in tissues of RSA patients. (a) By analyzing the GEO datasets (GSE113790), the expression level of ZBTB24 was found to be significantly downregulated in decidua tissues of the RSA patient as compared to the induced abortion controls (n = 3). (b) ZBTB24 expression level was quantified by RT-qPCR in placental villi of RSA patients (n = 15) and non-RSA patients undergoing abortion (n = 15). (c) & (d) Protein level of ZBTB24 in placental villi of RSA patients and non-RSA patients was examined by Western blot and immunohistochemistry (IHC) staining in 5 randomly selected samples in each group. **p < 0.01 and ***p < 0.001.

To investigate the implication of ZBTB24 in RSA patients, we first compared the ZBTB24 expression profile based on the data from GEO dataset (GSE113790), which contained 3 RSA samples (RSA) and three induced abortion samples (Control). The results revealed that ZBTB24 expression was significantly lower in the decidua tissues of the RSA patient as compared to the induced abortion controls (Figure 1(a)). To confirm the above findings, we further analyzed ZBTB24 expression level in the placental villi tissues of RSA patients and non-RSA patients with abortion (n = 15 in each group). RT-qPCR analysis showed that ZBTB24 expression level was significantly reduced in the placental villi tissues of the RSA patients (Figure 1(b)). The reduced expression of ZBTB24 was further confirmed at protein level by Western blot as well as Immunohistochemistry (IHC) staining (Figure 1(c,d)). These results suggest that ZBTB24 downregulation in the decidua and the placental villi tissues may be implicated in the progression of the RSA patients.

## ZBTB24 silencing inhibits the proliferation and induces the apoptosis of trophoblasts

**Figure 2.**
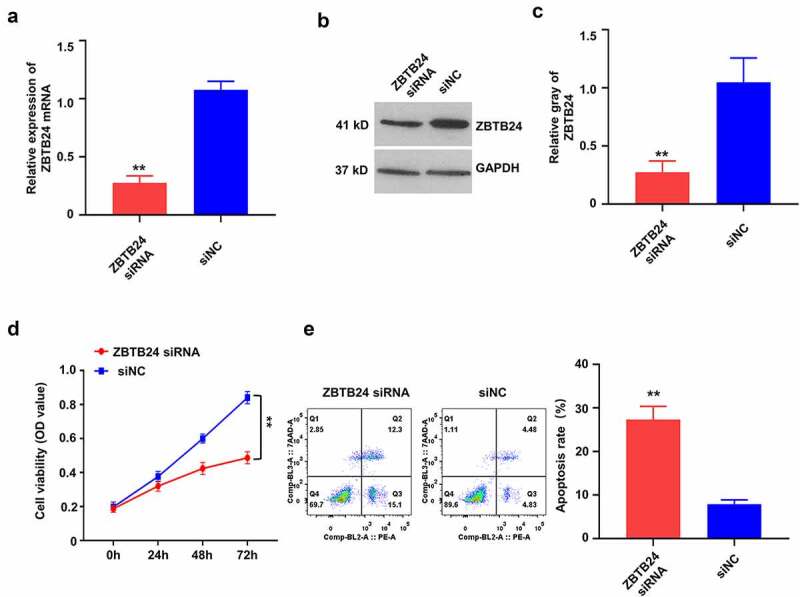
ZBTB24 silencing inhibits the proliferation, but promotes the apoptosis of trophoblasts. ZBTB24 silencing was performed by transfecting the HTR-8/SVneo cells with siRNA targeting ZBTB24. (a) RT-qPCR analysis of ZBTB24 expression after siRNA transfection. (b) & (c) Protein level of ZBTB24 was detected by Western blot assay after siRNA transfection (B, blot images; C, quantification). (d) CCK-8 cell proliferation assay and (e) apoptosis assay in the HTR-8/SVneo cells transfected with siRNA targeting ZBTB24. **p < 0.01.

To investigate the functional role of ZBTB24, we applied siRNA targeting ZBTB24 in HTR-8/SVneo cells. The silencing effect of ZBTB24 siRNA was confirmed at both the mRNA level by RT-qPCR (Figure 2(a)) and the protein level by Western blot (Figure 2(b,c)). We then investigated cell viability and apoptotic cell death after ZBTB24 silencing. The knockdown of ZBTB24 (ZBTB24-siRNA) significantly impaired cell proliferation when compared to control siRNA (siNC) (Figure 2(d)). Flow cytometry-based apoptosis detection revealed that ZBTB24 knockdown-induced apoptosis of the HTR-8/SVneo cells (Figure 2(e)). Together, our results suggest that ZBTB24 is required for the proliferation and survival of HTR-8/SVneo cells.

## Overexpression of ZBTB24 promotes cell proliferation and suppresses the apoptosis of trophoblasts

**Figure 3.**
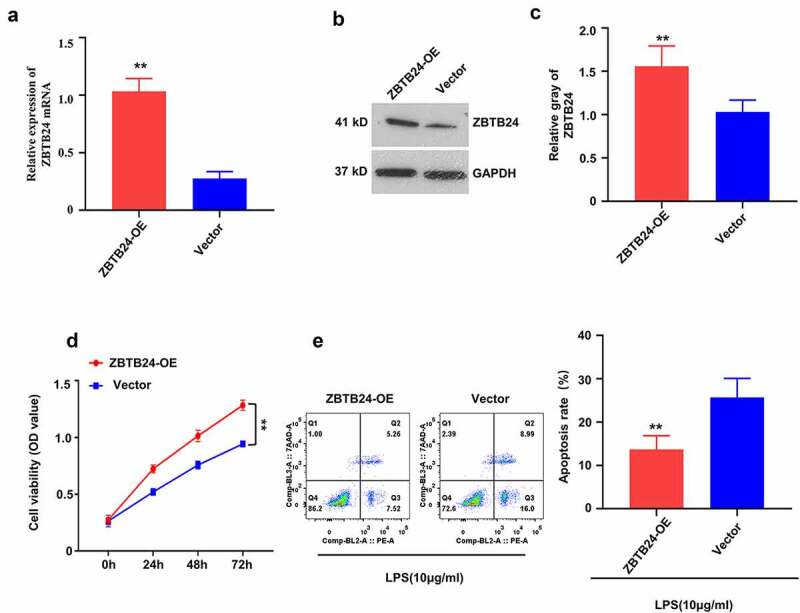
Overexpression of ZBTB24 promotes the proliferation, while inhibits the apoptosis in trophoblasts. To overexpress ZBTB24, pcDNA3.1-ZBTB24 plasmid was transfected into HTR-8/SVneo cells. (a) mRNA level of ZBTB24 was analyzed by RT-qPCR assay. (b) & (c) Protein level of ZBTB24 was examined by Western blot assay (B, blot images; C, quantification); (d) CCK-8 cell proliferation assay in ZBTB24 overexpression (ZBTB24-OE) and the control (vector) HTR-8/SVneo cells. (e) Apoptosis assay in ZBTB24 overexpression (ZBTB24-OE) and the control (vector) HTR-8/SVneo cells with 100 ng/ml LPS treatment for 24 h. **p < 0.01.

To further corroborate that ZBTB24 contributes to the proliferation and survival of trophoblasts, HTR-8/SVneo cells were transfected with ZBTB24 expression vector (pcDNA3.1- ZBTB24). Overexpression of ZBTB24 (ZBTB24-OE) was confirmed at both the mRNA level (Figure 3(a)) and the protein level (Figure 3(b,c)). CCK-8 proliferation assay demonstrated that ZBTB24 overexpression promoted cell proliferation (Figure 3(d)). To assess its effect in cell survival, we treated the cells with Lipopolysaccharides (LPS) to mimic inflammatory cell death. The overexpression of ZBTB24 significantly attenuated LPS-induced apoptosis in HTR-8/SVneo cells (Figure 3(e)). Collectively, our results show that ZBTB24 overexpression can promote the proliferative growth of HTR-8/SVneo cells.

## ZBTB24 promotes the syncytium formation ability of the trophoblasts

The formation of multinucleated syncytium from the mononucleate villous trophoblasts is associated with a decreased E-cadherin expression [7]. As a transient organ to mediate the maternal–fetal exchange, the secretion of pregnancy hormones (such as beta human chorionic gonadotropin β-hCG) and the placental development depends on the proliferation and differentiation of cytotrophoblasts [7,9].

**Figure 4.**
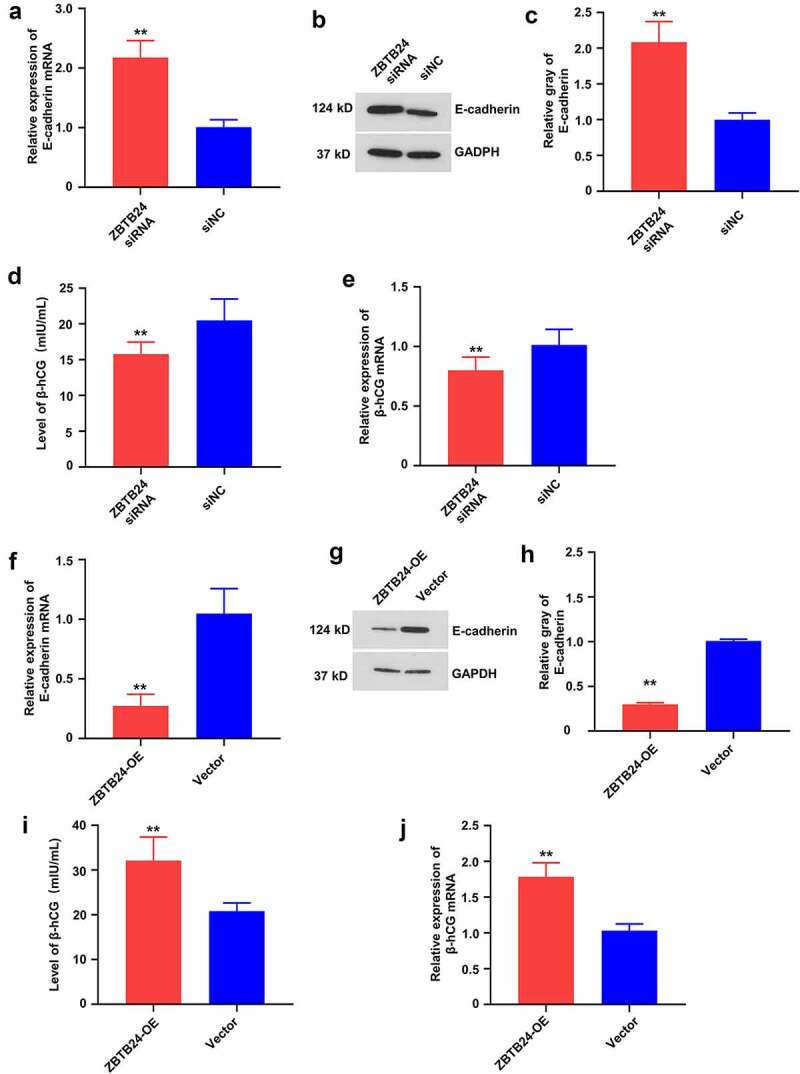
ZBTB24 regulates the expression of E-cadherin and β-hCG in trophoblasts. (a) mRNA level of E-cadherin was analyzed by RT-qPCR assay after ZBTB24 siRNA transfection. (b) & (c) Protein level of E-cadherin was examined by Western blot after ZBTB24 siRNA transfection. (d) Cell culture supernatant level of β-hCG was determined by ELISA after ZBTB24 siRNA transfection. (e) mRNA level of β-hCG was measured by RT-PCR after ZBTB24 siRNA transfection. (f) mRNA level of E-cadherin was analyzed by RT-qPCR after ZBTB24 overexpression. (g) & (h) Protein level of E-cadherin was examined by Western blot after ZBTB24 overexpression. (i) Cell culture supernatant level of β-hCG was determined by ELISA after ZBTB24 overexpression. (j) mRNA level of β-hCG was measured by RT-PCR after ZBTB24 overexpression. **p < 0.01.

To explore the implication of ZBTB24 in trophoblast differentiation, the expression levels of E-cadherin and β-hCG were examined in the HTR-8/SVneo cells after ZBTB24 knockdown (Figure 4(a-e)) or overexpression (Figure 4(f,j)). ZBTB24 knockdown significantly upregulated the mRNA and protein level of E-cadherin in the HTR-8/SVneo cells (Figure 4(a-c)). Meanwhile, the secretion (Figure 4(d)) and mRNA expression level (Figure 4(e)) of β-hCG were both suppressed after ZBTB24 knockdown. In contrast, the overexpression of ZBTB24 in HTR-8/SVneo cells significantly reduced the mRNA and protein levels of E-cadherin (Figure 4(f-h)), while the increased secretion (Figure 4(i)) and mRNA expression level (Figure 4(j)) of β-hCG were observed after overexpression of ZBTB24. Collectively, these data suggest that ZBTB24 could regulate the differentiation of trophoblasts

## ZBTB24 expression level regulates the migratory ability of the trophoblasts

**Figure 5.**
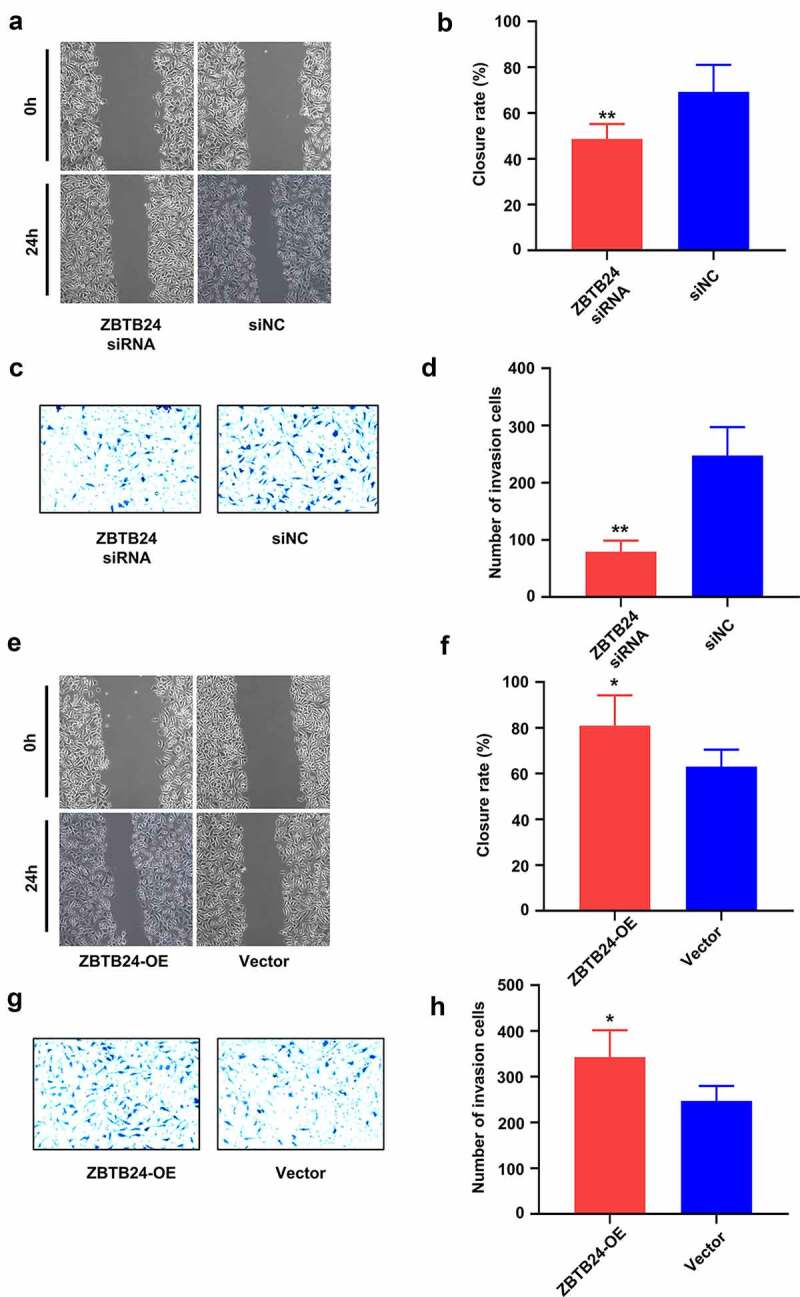
ZBTB24 promotes the migration of trophoblasts. (a) & (b) Wound-healing assay and (c) & (d) transwell migration assay was performed in HTR-8/SVneo cells after ZBTB24 siRNA transfection. (e) & (f) Wound-healing assay and (g) & (h) transwell migration assay was performed in HTR-8/SVneo cells after ZBTB24 overexpression. *p < 0.05, **p < 0.01.

Migration is an important characteristic of trophoblasts during placental formation [7,8]. To explore the functional role of ZBTB24 in trophoblast migration, we performed wound-healing assay and transwell migration assay in the HTR-8/SVneo cells with ZBTB24 silencing or knockdown. ZBTB24 silencing significantly inhibited the migration ability of the HTR-8/SVneo cells in both would healing and transwell migration assay (Figure 5(a-d)). In contrast, ZBTB24 overexpression in HTR-8/SVneo cells promoted their migratory ability (Figure 5(e-h)). Therefore, ZBTB24 expression level could positively regulate the migratory ability of the trophoblasts.

## ZBTB24 regulates E-cadherin expression by modulating the DNA methylation at the promoter region

**Figure 6.**
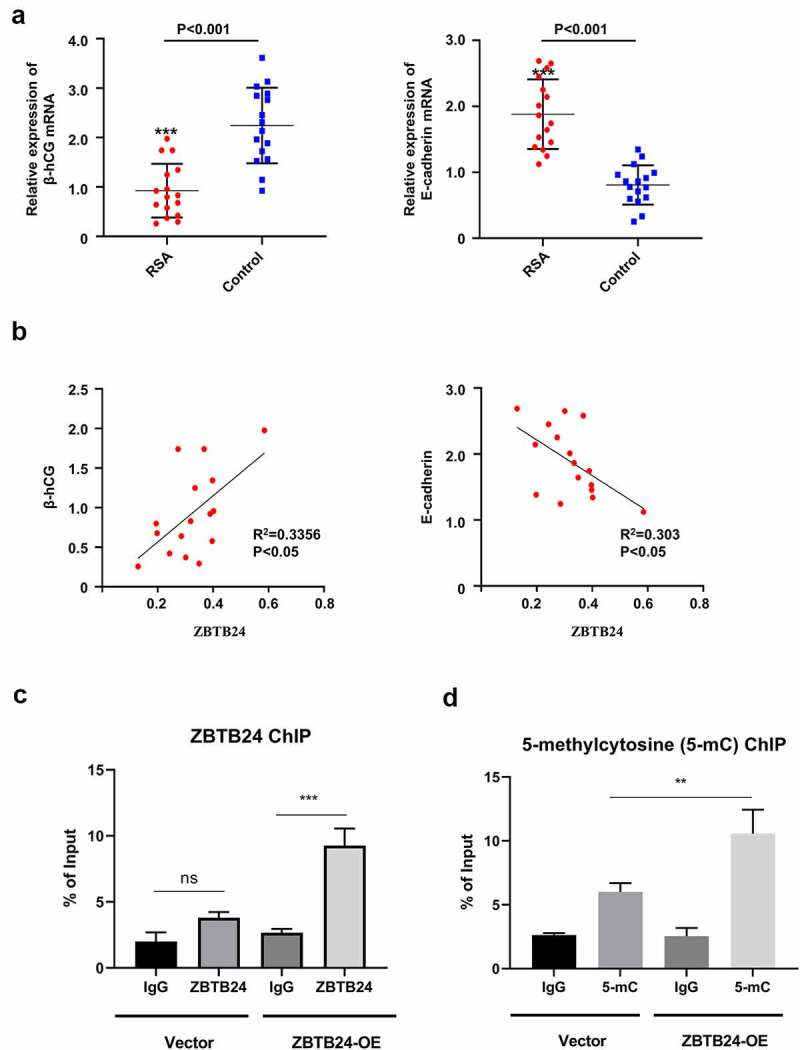
ZBTB24 regulates E-cadherin expression by modulating the DNA methylation at its promoter. (a) mRNA level of β-hCG and E-cadherin was measured by RT-PCR in placental villi of RSA patients (n = 15) and non-RSA patients undergoing abortion (n = 15). (b) Spearman correlation analysis between ZBTB24 and β-hCG expression level, and the correlation between ZBTB24 and E-cadherin level. (c) ZBTB24 ChIP analysis in ZBTB24 overexpression (ZBTB24-OE) and the control (vector) HTR-8/SVneo cells. (d) 5-methylcytosine (5-mC) ChIP analysis in ZBTB24 overexpression (ZBTB24-OE) and the control (vector) HTR-8/SVneo cells. IgG isotype antibody was used as negative control for ChIP. **p < 0.01 and ***p < 0.001.

To further show the implication of ZBTB24 in regulating E-cadherin and β-hCG in RSA, we analyzed their expression in the RSA and control samples. The data reveals that β-hCG was reduced in RSA samples while E-cadherin was upregulated in RSA samples (Figure 6(a)). Importantly, Spearman correlation analysis revealed a positive correlation between ZBTB24 and β-hCG expression level, and a negative correlation between ZBTB24 and E-cadherin level (Figure 6(b)). Since ZBTB24 can coordinate with DNMT3B to regulate DNA methylation [13–15], we further investigate whether ZBTB24 suppresses E-cadherin expression by modulating the DNA methylation at its promoter by chromatin immunoprecipitation (ChIP) assay. We first compared the binding of ZBTB24 to E-cadherin promoter region in ZBTB24 overexpressing and control cells, using a primer set spanning a region 200 bp upstream of the start codon). ZBTB24 ChIP analysis showed that ZBTB24 was specifically enriched at E-cadherin promoter when ZBTB24 was overexpressed (Figure 6(c)). We further analyzed the level of DNA methylcytosine (5-mC) using an anti-5-methylcytosine (5-mC) antibody. ChIP analysis showed when ZBTB24 was overexpressed in HTR-8/SVneo cells, 5-mC level was significantly enhanced at E-cadherin promoter. Collectively, these data indicate that ZBTB24 could bind to E-cadherin promote and modulate the DNA methylation.

## Discussion

Cell fusion is required for the formation of multinucleated syncytium from trophoblasts, which is crucial for the gestation, such as placentation, fertilization, and fetal development [17]. As a transient organ, human placenta is responsible for maternal–fetal exchange of nutrients, elimination of wastes, secretion of hormones, and the immunologic protection of the fetus [17–20]. The malfunction of trophoblasts could account for the RSA [7,8]. In this study, we revealed that ZBTB24 is downregulated in the decidua and the placental villi tissues of the RSA patients, suggesting a potential implication of ZBTB24 in the pathogenesis of RSA. We further performed loss-of-function and gain-of-function experiment to demonstrate the necessity and requirement of ZBTB24 in regulating the functions of trophoblasts, such as proliferation, cell survival, and migratory capability. The observation that ZBTB24 knockdown significantly inhibits the proliferative and migration potentials of trophoblast highlights the indispensable role of ZBTB24 in controlling the phenotype of trophoblast. These findings also indicate that the downregulation of ZBTB24 in the placental villi tissues may lead to the malfunction of trophoblasts in RSA patients.

The development of placental structures, such as floating villi and anchoring villi, depends on the differentiation and proliferation of trophoblasts during pregnancy [21–23]. Cadherin family members play central roles in trophoblast differentiation and placentation [24]. Among them, E-cadherin expression is spatiotemporally regulated and reduced during the morphological and functional differentiation of human mononucleate villous cytotrophoblasts into the multinucleated syncytium [25]. E-cadherin expression is lost during the syncytialization of cytotrophoblasts, while its expression level remains high in extravillous trophoblasts [26]. Our data also show that ZBTB24 expression level regulates E-cadherin expression. Silencing ZBTB24 upregulates E-cadherin, while ZBTB24 overexpression reduces E-cadherin level. Therefore, these data suggest that ZBTB24 may control trophoblast phenotypical and functional differentiation by regulating E-cadherin expression.

As a member of the Bric-à-brac/poxvirus and zinc finger (BTB/POZ) family, ZBTB24 could coordinates with DNMT3B (DNA Methyltransferase 3 Beta) to modulate DNA methylation [13,14]. We further performed ChIP assay and demonstrated that ZBTB24 could bind to E-cadherin promoter and induce the hypermethylation, which underlies the downregulation of E-cadherin by ZBTB24 overexpression. This finding is also consistent with the previous observation that abnormal DNA methylation is associated with karyotypically normal miscarriage reported in women suffering from RSA [11,12].

## Conclusion

In conclusion, the current work uncovered that ZBTB24 was significantly downregulated in placental tissues of RSA patients. The reduced level of ZBTB24 may inhibit the proliferation and migration of trophoblasts, and impairs the functional differentiation of trophoblast by modulating E-cadherin expression. Our findings highlight the potential functional role of ZBTB24 in placental trophoblasts. Future work needs to focus on the investigation of the mechanisms by which ZBTB24 is downregulated in the placental tissues of RSA patients.

## Data Availability

The authors declare that all supporting data are available in this article.

## Abbreviations

RSA: recurrent spontaneous abortion; SFM: serum free media; FBS: fetal bovine serum; BCA: bicinchoninic acid assay; PVDF: polyvinylidene fluoride; cDNA: complementary deoxyribonucleic acid; GAPDH: Glyceraldehyde-3-Phosphate Dehydrogenase; RT-qPCR: real-time quantitative PCR; siRNA: short interference RNA; SDS-PAGE: polyacrylamide gel electrophoresis; HRP: horseradish peroxidase; β-hCG, human chorionic gonadotropin.

## Highlights

- ZBTB24 expression was downregulated in clinical samples of RSA patients;
- ZBTB24 level positively regulates cell viability and migration of trophoblasts;
- ZBTB24 regulates E-cadherin expression by modulating DNA methylation at promoter.
